# Genetic Connectivity in Scleractinian Corals across the Northern Gulf of Mexico: Oil/Gas Platforms, and Relationship to the Flower Garden Banks

**DOI:** 10.1371/journal.pone.0030144

**Published:** 2012-04-30

**Authors:** Paul W. Sammarco, Daniel A. Brazeau, James Sinclair

**Affiliations:** 1 Louisiana Universities Marine Consortium (LUMCON), Chauvin, Louisiana, United States of America; 2 Department of Oceanography and Coastal Sciences, A&M College, Louisiana State University, Baton Rouge, Louisiana, United States of America; 3 Pharmaceutical Genetics Laboratory, Department of Pharmaceutical Sciences, University at Buffalo, Buffalo, New York, United States of America; 4 Environmental Section, Bureau of Ocean Energy Management, Regulation, and Enforcement (BOEMRE), Gulf of Mexico OCS Region and Atlantic Activities, United States Department of the Interior, New Orleans, Louisiana, United States of America; University of Canterbury, New Zealand

## Abstract

The 3,000 oil/gas structures currently deployed in the northern Gulf of Mexico (GOM) provide hard substratum for marine organisms in a region where such has been rare since the Holocene. The major exception to this are the Flower Garden Banks (FGB). Corals are known to have colonized oil/gas platforms around the FGB, facilitating biogeographic expansion. We ask the question, what are the patterns of genetic affinity in these coral populations. We sampled coral tissue from populations of two species occurring on oil and gas platforms: *Madracis decactis* (hermatype) and *Tubastraea coccinea* (invasive ahermatype). We sampled 28 platforms along four transects from 20 km offshore to the continental shelf edge off 1) Matagorda Island, TX; 2) Lake Sabine, TX; 3) Terrebonne Bay, LA; and 4) Mobile, AL. The entire population of *M. decactis* was sampled between depths of 5 m and 37 m. *T. coccinea* populations were sub-sampled. Genetic variation was assessed using the PCR-based Amplified Fragment Length Polymorphisms (AFLPs). Data were analyzed via AFLPOP and STRUCTURE. Genetic connectivity among *M. decactis* platform populations was highest near the FGB and decreased to the east. Connectivity increased again in the eastern sector, indicating isolation between the populations from different sides of the Mississippi River (Transects 3 and 4). A point-drop in genetic affinity (relatedness) at the shelf edge south of Terrebonne Bay, LA indicated a population differing from all others in the northern GOM. Genetic affinities among *T. coccinea* were highest in the west and decreased to the east. Very low genetic affinities off Mobile, AL indicated a dramatic difference between those populations and those west of the Mississippi River, apparently a formidable barrier to larval dispersal.

## Introduction

Prior to the 1940s, the bottom of the Gulf of Mexico (GOM) was characterized primarily by terrigenous, sandy muds with low habitat diversity [1], [2]. During that decade, offshore drilling for oil and gas began there and production platforms grew steadily in number, spreading southward across the continental shelf. Those platforms served as substrate for colonization of numerous marine organisms, and this process has continued [3]–[8]. These production platforms extend up from the bottom into the atmosphere, creating an island and providing hard substrate through all depths of the water column [9] that would otherwise not be available to benthic or demersal marine organisms. It has been estimated that a 200 ft. tall platform jacket can provide acres of hard substrate, supporting algae, barnacles, mussels, and other sessile epibenthic invertebrates [2], [4]. In earlier studies, we and others have documented the presence of both hermatypic (zooxanthellate, reef-building) and ahermatypic (azooxanthellate, non-reef-building) scleractinian corals on many of these platforms [5], [10]–[17].

**Figure 1:**
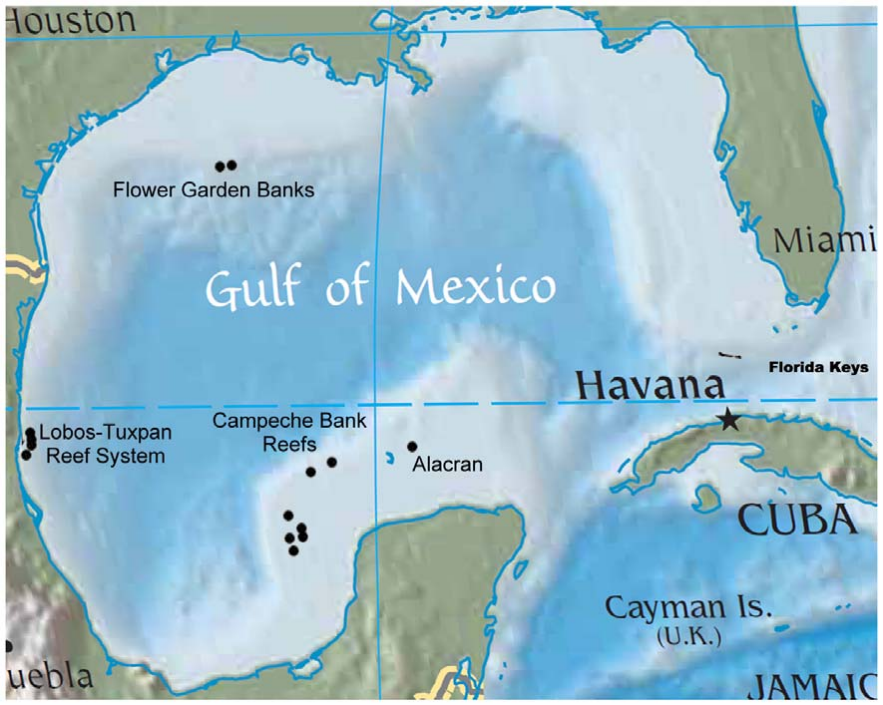
Map of the Gulf of Mexico depicting the location of the Flower Garden Banks and their nearest neighboring major natural reefs, e.g., the Lobos-Tuxpan reef system, Campeche Bank reefs, Alacran, and the Florida Keys.

The only true coral reefs in the northern Gulf of Mexico are the Flower Garden Banks (FGB; NOAA Flower Garden Banks National Marine Sanctuary) [18], located ∼180 km SE of Galveston, TX. The FGB are defined by two banks that approach the surface to within 18 m [19]: the East Bank (27°54′32′′ N, 93°36′ W) and West Bank (27°52′27′′ N, 93°48′47′′ W) [1], [20] (see Fig. 1). Calcium carbonate reefs have developed on their caps [20], [21] which are productive [22] and healthy, being characterized by 24 species of hermatypic corals) [18]–[20], [23]. The closest reefs to the FGB are the Lobos-Tuxpan system, located 13 km off Cabo Rojo, Mexico [18] (Fig. 1), ≥640 km away [24], [25]. Other banks do exist on the northwestern GOM shelf, such as Stetson, Sonnier, 28 Fathom, etc. and do possess scleractinian corals [19], [22], [25]–[29]. These banks are deeper, however, or occur in cooler waters and do not qualify as true coral reefs because they are not biogenic in origin (*i.e.,* composed of calcium carbonate that has been accreted by corals). The FGB are now surrounded by hundreds of platforms. It is possible that coral populations on the deeper banks could be a source of larvae that might colonize the platforms, but the abundance of coral on those banks is much lower than those on the FGB. The potential of the banks being a larval source for recruitment for the platforms in is probably relatively low.

In this study, we focused on one hermatypic (zooxanthellate and reef-building) scleractinian coral species and one ahermatypic (azooxanthellate and non-reef building) one which occur on the platforms in the northern GOM and also on the FGB. We attempted to determine the degree of genetic connectivity (or relatedness) among the natural and platform populations on a large geographic scale, covering most of the northern GOM. Through earlier surveys, we found that these species were abundant enough to provide sample sizes sufficient for meaningful comparative molecular genetic analysis. The corals were *Madracis decactis* (Lyman 1859; Pocilloporidae; hermatype) and *Tubastraea coccinea* (Lesson 1829; Dendrophylliidae; ahermatype). Both of these reproduce via brooding. *Madracis decactis* is a simultaneous hermaphrodite and planulates monthly between March and December, with a broad peak occurring from Sept. to Nov. [30]. *Tubastraea coccinea* produces planulae sexually [31] but can also produce them asexually [32], [33]. It commonly produces numerous runners and is highly effective at producing new colonies asexually [34], [35]. This invasive species was first observed to colonize the East Flower Garden Bank in 2002 [36].

*Tubastraea coccinea* is the single, most successful invasive coral and one of the most successful of all species to invade the Atlantic Ocean [37]–[40]. Other known invasive corals in the Caribbean are *Fungia scutaria* [41]–[43] and now *Tubastraea micranthus* (Ehrenberg 1834), a recent introduction to the region just south of the Mississippi River mouth (the Grand Isle – GI- lease area) [44]. Figueira de Paula and Creed [45] have also reported the introduction of *T. tagusensis* to Brazil, along with that of *T. coccinea.* Thus, the total number of introduced coral species to the western Atlantic Ocean is now four. *T. coccinea* was first recorded in Puerto Rico in 1943 and then in Curacao, Netherlands Antilles in 1948, occurring on ships’ hulls [46]. It appeared in Belize and Mexico in the late 1990s and early 2000s [37]. Its spread progressed to Venezuela, northern Gulf of Mexico, and the Florida Keys [25], [38], [39]; Brazil [45]; and Colombia, Panama, the Bahamas, and throughout the Lesser and Greater Antilles [40]. In terms of sheer numbers, *Tubastraea coccinea* is clearly the most abundant scleractinian coral, hermatypic or ahermatypic, in the northern Gulf of Mexico on artificial substrata [14]–[16], [25], [47]. Hundreds of thousands of colonies may be found on a single platform (e.g., 28/m^2^) [14], [48]. It has also been found on some of the deeper banks of the northern GOM, but its abundances are low there [26], [28], [49]. It was first observed to colonize the East Flower Garden Bank in 2002 [36]. Its abundances there are also low, where it only occurs cryptically. It would appear that *T. coccinea* is not as successful a competitor for space on natural well-established reefs as on artificial substrate.

The two target coral species are brooders and reproduce by producing fully developed larvae which have the capability to settle in ≥4hrs [50]. Recruits can become reproductively mature within 1–2 yrs of age. Brooders generally planulate on a monthly cycle and can release larvae up to 8–10 times per year (e.g., *Porites astreoides*) [51]. Planulae are released during a spawning event that may extend over a period of days. Data from some coral studies suggest that brooders are adapted for short-distance dispersal. Broadcast spawners (corals that release sperm and eggs into the water column for fertilization and larval development there), on the other hand, are believed to be more effective at longer-distance dispersal (see [52]). The potential for long-range dispersal between planulae produced by these two types of corals (brooders and broadcasters) is most likely comparable once the planulae have become fully developed [53], [54].

**Figure 2:**
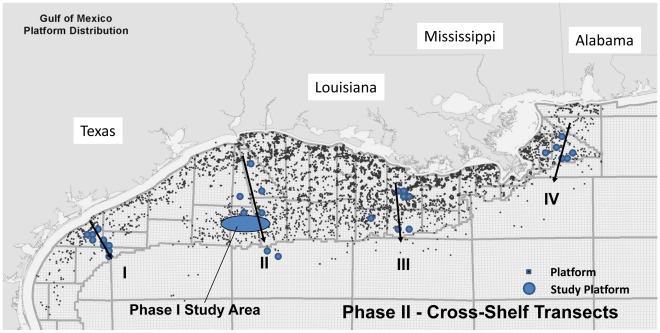
Map of the oil and gas platforms in the northern Gulf of Mexico. Four cross-shelf transects (I – IV, west to east) were used to examine scleractinian corals on a sub-set of oil/gas production platforms along each transect. Small squaresrepresent platforms. Large dots represent study platforms sampled for corals; see Table 1 for specific names, latitudes, longitudes, and lease area names of platforms. The transects ran generally SE from Matagorda Island, Texas; S from Port Arthur/Lake Sabine, LA; S from Timbalier Island, LA; and SW from Mobile, Alabama. The oval represents a region encompassing the Flower Garden Banks and 13 platforms sampled in an earlier study for coral community development and population genetics [25].

Here we examine genetic affinities among populations of the scleractinian corals *Madracis decactis* and *Tubastraea coccinea,* respectively. The populations sampled werefrom the Flower Garden Banks and a large number of platforms across the continental shelf of the northern Gulf of Mexico. The platforms cover ∼800 km of coastline from Matagorda Island, TX to Mobile, AL. The FGB occur west of the center of this range. We will examine the degree of connectivity between coral populations on the platforms in this region, and between those platforms and the natural reefs. We will also attempt to infer information about the comparative effectiveness of dispersal and colonization by these two brooding species, and relate it to an island-hopping strategy of colonization as described by MacArthur and Wilson ([55], also see [52], [56]–[58]). We will also expand the original analyses of Atchison et al. [58], [59], who conducted similar studies on *Madracis decactis* and *Diploria strigosa* (a broadcasting coral) in this region. We also utilize a more conservative statistical analytical approach to analyze the data, based on extensive simulations. The specific objectives of this study are: To determine the degree of genetic connectivity between adult coral populations of *Madracis decactis* (hermatype) and of *Tubastraea coccinea* (ahermatype) on oil/gas platforms throughout the northern Gulf of Mexico and the Flower Garden Banks; to compare variation in genetic affinity between conspecific populations on different platforms; to determine the degree of genetic affinity between conspecific populations on the platforms *vs.* the FGB; and, to utilize data on patterns of genetic variation to infer comparative colonization potentials for these species.

**Table 1:** Details of the oil and gas platforms studied in the northern Gulf of Mexico along four cross-continental shelf transects from Matagorda Island, Texas to Mobile, Alabama.

					Gulf of Mexico						
Far Western Sector (I)			Western Sector (II)			Central Sector (III)				Near Eastern Sector (IV)	
Platform	lat.	long.	Platform	lat.	long.	Platform	lat.	long.	Platform	lat.	long.
**MI-651-A**	28.0222	−96.3071	**WC-312-1**	29.1872	−93.5822	**ST-165-A**	28.5767	−90.5769	**MP-159-1**	29.6491	−88.4647
**MI-618-A**	28.0222	−96.3071	**WC-414-A**	28.7579	−93.3878	**ST-163-A**	28.5720	−90.4996	**MP-236-B**	29.4054	−88.5844
**MI-672-A**	27.9942	−96.2596	**HI-A-237-A**	28.6678	−93.8857	**ST-188-CA**	28.5010	−90.3808	**MP-265-A**	29.3467	−88.2816
**MI-672-B**	27.9688	−96.2909	**WC-522-A**	28.3759	−93.4912	**ST-190-A**	28.4663	−90.4461	**MP-144-A**	29.2924	−88.6691
**MI-A-4A**	27.9042	−96.0892	**HI-A-287-A**	28.3610	−93.7690	**SS-277-A**	28.2993	−91.0876	**MP-289-B**	29.2585	−88.4415
**BA-104-A**	27.8669	−96.0334	**GB-189-A**	27.7786	−93.3095	**ST-292-A**	28.2141	−90.4203	**MP-288-A**	29.2398	−88.4095
**BA-A-133-A**	27.8545	−96.0364	**GB-236-A**	27.7611	−93.1377	**ST-295-A**	28.1963	−90.5413			
**BA-133-D**	27.8388	−96.0282									
**Lease Area**											
**Abbrev.**	**Name**										
**BA**	Brazos										
**GB**	Garden Banks										
**HI**	High Island										
**MI**	Matagorda Island										
**MP**	Main Pass										
**SS**	Ship Shoals										
**ST**	South Timbalier										
**WC**	West Cameron										

Platforms served as study sites for coral collection. Names of platforms shown, along with their latitudes and longitudes. Platforms are listed from north to south (by latitude); see Fig. 2 for graphic representation of location. Definitions of abbreviations designating offshore lease areas also shown.

## Materials and Methods

### Study Site

We initiated a set of field surveys followed by laboratory analyses. Surveys were conducted by SCUBA on offshore platforms extending between Madagorda Island, TX and Mobile, AL – a distance of 780 km. We sampled an area from ∼20 km offshore to the edge of the continental shelf and beyond. Platforms were sampled along four cross-shelf transects spaced at approximately equal intervals along the GOM coast (Fig. 2; Table 1). Transect 1 ran SE from Matagorda Island, Texas and included 8 platforms; Transect 2 ran S from Port Arthur/Lake Sabine, LA (7 platforms); Transect 3 ran S from Timbalier Island, LA (7 platforms); and Transect 4 ran SW from Mobile, Alabama (6 platforms).

These transects were chosen, firstly because they covered the breadth and width of the shelf where platforms exist. particularly the shelf edge. Secondly, they cover enough of the shelf to potentially provide northern boundary information regarding coral colonization and survival. In addition, they would provide information on coral colonization and growth on platforms near the shelf edge.

### Sample Collection

We chartered a dive vessel (M/V *Fling*, Freeport, Texas) and conducted surveys over a period of three years (Fig. 2; Table 1). Surveys were conducted between 5 and 37 m depth with teams of SCUBA divers during the summer and fall seasons of 2004–2007. All platforms surveyed had been deployed for 15–26 years, since it has been determined that a minimum of 15 yrs is associated with the development of substantial adult coral populations [25]. (Data on distribution, abundance, and species diversity of corals in this region may be found in a previous publication) [47]. At the East and West FGB, coral tissue samples were collected by SCUBA divers haphazardly.

Tissue samples, two cm^2^ in area, were collected by SCUBA divers from the growing edge of adult corals of the two target species using small hammers and chisels. Tissue was collected from all *Madracis decactis* coral colonies encountered on the platforms, representing a total population sample for these depths. Total population sizes of *Madracis decactis* on the platforms were small. On the other hand, *Tubastrea coccinea* was very abundant, and thus sub-samples were taken (see Table 2 for summary). Only sites supplying a sufficient number of corals were considered in our analyses, to reduce potential confounding effects due to low sample size. No specific permits were required for the described field studies. Permission was obtained from the oil and gas companies to dive on and collect specimens from their platforms.

**Table 2:** A list of platforms sampled and number of coral colonies sampled per platform in each transect/sector across the northern Gulf of Mexico.

Species	Transect Number/Sector	Platform	No. Coral Samples
***Tubastraea***	**Transect 1**	BA-133-A	*16*
***coccinea***	**Western Sector**	MI-A4-A	48
	
	**Transect 2**	GB-189-A	*31*
	**Near Western Sector**	GB-236-A	7
		HI-237-A	8
		HI-287-A	8
		WC-522-A	14
		W-FGB	19
	**Transect 3**	ST-188-A	14
	**Central Sector**	ST-190-A	14
		*ST-262-A*	*0*
		ST-277-A	14
		ST-292-A	*17*
		ST-295-A	*19*
	**Transect 4**	MP-144-A	*16*
	**Near Eastern Sector**	MP-236-B	12
		MP-265-A	*46*
		MP-288-A	*27*
		MP-289-B	31
***Madracis***	**Transect 2**	GB-236	*14*
***decactis***	**Near Western Sector**		
	**Transect 3**	ST-277-A	9
	**Central Sector**	ST-292-A	21
		ST-295-A	30
	**Transect 4**	MP-236-B	1
	**Near Eastern Sector**	MP-144-A	2
		MP-265-A	2
		MP-289-B	1
		Total	441
***No.*** ** = samples w/low or no DNA yield**			

Numbers are provided for two target coral species – *Madracis decactis* and *Tubastraea coccinea*. Data were combined in the Main Pass (MP) lease area in the case of the former species due to small sample sizes per platform. Inviable samples not included in analysis shown in italics.

The samples were returned to the ship and sealed in plastic freezer bags containing SED high-salt buffer (saturated NaCl, 250 mM EDTA, pH 7.5, 20% DMSO) to preserve the DNA. This preservative allows tissue samples to be stored at room temperature, eliminating the need for storage in liquid nitrogen. The samples were then placed in additional SED buffer, placed in ice chests, and returned to the laboratory.

### Amplified Fragment Length Polymorphisms (AFLPs)

Amplified Fragment Length Polymorphism (AFLP) is a DNA-“fingerprinting” technique [60] that detects polymorphisms based upon the selective PCR amplification of a subset of numerous restriction fragments generated by two different restriction enzymes [61], [62]. The AFLPs tend to be highly polymorphic, but they are not co-dominantly expressed. They are commonly used in studies of commercial crop species and other economically important species; but they have not been widely used in animal studies [63]. This is surprising, since AFLPs provide abundant polymorphic markers relatively quickly for any species of interest. AFLPs have been used successfully to estimate migration rates [64], species boundaries [65], [66], and degree of parental contributions to populations [67]. Use of AFLPs is not ideal for all population genetic applications [60]. They do, however, perform extraordinarily well for population assignment or allocation studies [62], [68]–[71], where the number of polymorphic loci is more important than allelic diversity [72]. In this case, 117 polymorphic markers were generated and utilized for the study. Only those samples yielding readable markers were included in the study, defining the sample sizes for each site (see Table 3 for a summary).

**Table 3:** Sequences of the adapters and primers used in the AFLP protocol.

	Name	Sequence
**Adapters EcoRI**	EcoF	5′-CTCGTAGACTGCCTACC
	EcoR	5′-AATTGGTACGCAGTCTAC
**Adapters MseI**	MseF	5′-GACGATGAGTCCTGAG
	MseR	5′-TACTCAGGACTCAT
**Pre-selective Primer**	EcoRI **A**	5′-GACTGCGTACC AATTC **A**
**Pre-selective Primer**	MseI **C**	5′-GATGAGTCCTGAG TAA **C**
**Selective Primers (Set 1)**	EcoRI	5′-GACTGCGTACCAATTC **ACT**
	MseI	5′-GATGAGTCCTGAGTAA **CAG**
**Selective Primers (Set 2)**	EcoRI	5′-GACTGCGTACCAATTC **ACC**
	MseI	5′-GATGAGTCCTGAGTAA **CTT**

(Pre-selective and selective nucleotides are indicated in bold.).

It is possible that some genetic variation detected using AFLPs may not be derived from the target organism [60]. This has been an area of concern with corals, which possess endosymbiotic zooxanthellae. Here, however, we have used zooxanthella-specific PCR primers to confirm for each sample that any contamination by zooxanthellar DNA occurs at levels far below those necessary for AFLP (*i.e.,* 5–10 pg of zooxanthellar DNA in a background of coral DNA) [69].

### Preparation of Coral Tissue Lysates for Genetic Analysis

DNA was isolated by macerating samples lightly in SED buffer and spinning at 16Xg for 5 min to pellet the zooxanthellae from the homogenate. The DNA was then purified using the Wizard® SV Genomic DNA Purification System (Promega Corporation, Madison, WI), following the manufacturer’s instructions for animal tissue. All samples were checked for zooxanthellae DNA contamination using the PCR techniques described in Brazeau et al. [69] and Atchison et al. [58], [59].

The AFLP technique, like other similar molecular genetic techniques, generate a subset of markers from a large population of markers. Of the subset obtained from a given AFLP experiment, a portion is often sensitive to specific reaction conditions. Thus, extra caution is required in processing samples through all procedural steps to maximize repeatability of results. Here, we processed samples in large lots containing members from all populations. This helped to distribute any error potentially introduced by reaction conditions uniformly between populations in an unbiased fashion. Also, all PCR reactions were replicated using one machine and the same thermal cycle profiles.

### Genomic Coral DNA digestion and adapter ligation

A restriction-ligation “master mix” was prepared using the following reagents (measures are per sample): 1.1 µl T_4_ DNA ligase 10X buffer (30 mM Tris-HCl, pH 7.8/10 mM MgCl_2/_10 mM dithiothreitol (DTT )/1 mM ATP), 1.1 µl of 0.5 M NaCl, 0.5 µl bovine serum albumin (BSA; 1 mg/ml), 1.0 µl Mse I adapters (50 µM), 1.0 µl EcoRI adapter (5 µM), 0.25 µl Mse I (4 U/µl; New England BioLabs, Beverly, MA), 0.25 µl of EcoRI (20 U/µL; New England BioLabs), and 0.33 µl of T_4_ ligase (3 U/µl; 10 mM Tris-HCl, pH 7.0/50 mM KCl/1 mM DTT/0.1 mM EDTA/50% glycerol). Sequences for the Mse I and EcoRI adapters and PCR primers are listed in Table 3. To each new 1.7 ml tube, 5.5 µl of the restriction-ligation mixture plus 5.5 µl (500 ng genomic DNA) of the purified genomic was added, centrifuged for 15 s, and incubated at room temperature overnight. At the end of the restriction-ligation reaction, 189 µl of TE buffer (10 mM Tris-HCl, pH 8.0/0.1 mM EDTA) was added (10-fold dilution), serving as the template for the next-step, pre-selective amplification.

**Figure 3:**
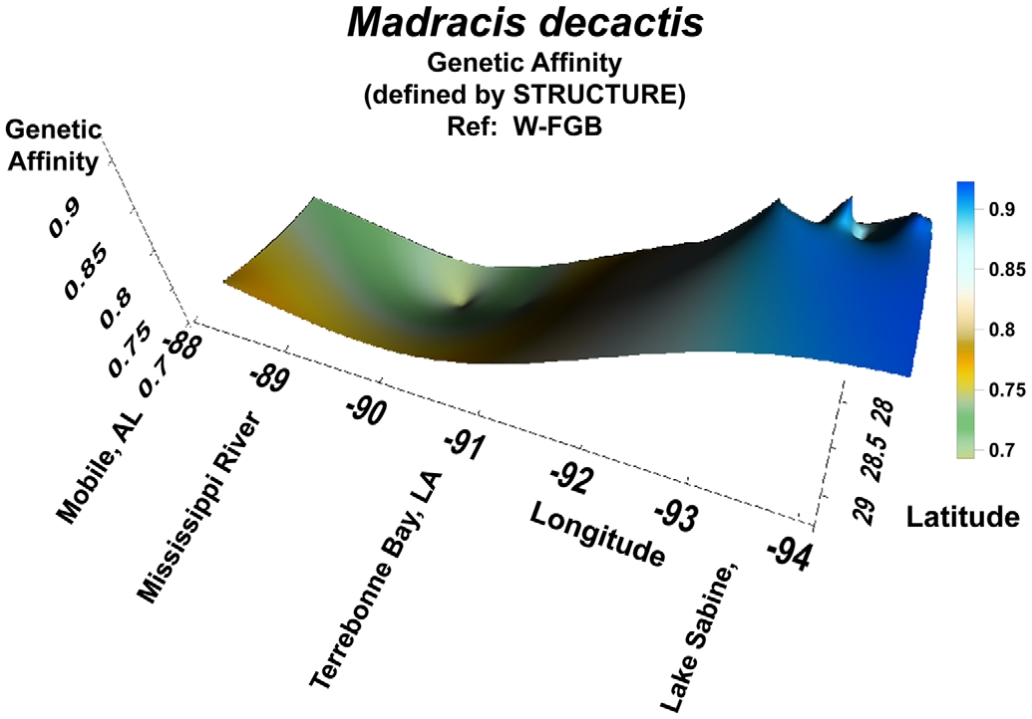
Genetic affinity in *Madracis decactis* coral populations on oil and gas platforms across the continental shelf in the northern Gulf of Mexico. Genetic affinity value (degree of relatedness) determined by the population genetics analytical software STRUCTURE. The reference population was the West Flower Garden Bank; *i.e.,* the population against which all other members of all other populations were compared The peak in the west implies that corals on platforms in that region were most likely derived from the Flower Garden Banks. Population differentiation is evident in the east, on either side of the Mississippi River mouth. The point-depression south of Terrebonne Bay, LA may represent a population drawn from outside of the northern Gulf of Mexico. Note: The orientation of the map has been reversed to east-to-west in order to facilitate viewing of the topography of the three-dimensional pattern generated by the data. This reveals fine-scale structure that would otherwise be hidden using a southerly view. The reader is viewing the region from the north, with east being on the left and west on the right.

### Pre-selective (PS) Amplification of Coral DNA

A second “master mix” was made for pre-selection (PS) amplification, using the following reagents (per sample measure given): 8.1 µl of nuclease-free water, 2.0 µl of 10X PCR buffer (15 mM Mg++ in buffer), 0.8 µl of 5 mM dNTP’s, 2.0 µl of EcoRI PS primer (2.75 µM), 2.0 µl of MseI PS primer (2.75 µM), and 0.1 µl of Thermostable (Taq) DNA polymerase (5 U/µl), for a total volume of 15.0 µl. Fifteen µl of the pre-selective amplification master mix was combined with 5 µl of each of the diluted restriction ligation reaction in a 0.5 ml tube. Samples were vortexed and centrifuged for 15 s. Amplification was performed using a 2-min initial incubation at 72°C, followed by 20 cycles of 20 s denaturation at 94°C, 30 s annealing at 56°C, and 2 min extension at 72°C. Last steps were 2 min final extension at 72°C, and 30 min final incubation at 60°C. After the cycling was completed, 180 µl of TE buffer was added to each tube, which consisted of the templates for the final step, selective amplification.

### Selective Amplification of Coral DNA

In the final step, a selective amplification “master mix” was made, containing the following components: 8.1 µl of nuclease-free water, 2.0 µl of 10X PCR buffer (with Mg++ at 15 mM), 0.8 µl of 5 mM dNTP’s, 2.0 µl of EcoRI selective primer (0.46 µM), 2.0 µl of MseI selective primer (2.75 µM), and 0.1 µl of Taq DNA polymerase (5 U/µl) for a total volume of 15.0 µl. To each 0.5 ml micro-centrifuge tube, 5 µl of the diluted pre-selection PCR reaction was added to each corresponding tube, mixed, and centrifuged for 15 s. Samples were placed in the thermocycler, and the cycle profile was performed as indicated: 2 min initial denaturation at 94°C, followed by 1 cycle of 20 s denaturation at 94°C, 30 s annealing at 66°C, and 2 min extension at 72°C. Next, there were 9 cycles: 20 s at 94°C, initial 30 s at 66°C (reduced 1°C/cycle), and 2 min at 72°C. Final cycle consisted of 20 cycles: 20 s at 94°C, 30 s at 56°C, and 2 min at 72°C, followed by a 30 min final incubation at 60°C.

Products for the selective PCR were run on an Amersham MegaBACE 1000 96 capillary sequencer at the University of Florida’s Interdisciplinary Center for Biotechnology Research. Resulting electropherograms were analyzed using SoftGenetics GeneMarker® (ver 1.51) for bands ranging from 50 to 400 bp in size in 20 bp increments.

The Selective PCR reactions were repeated three times for each sample. These “repeat” reactions were run on different days with populations mixed in each run. Bands were considered present if they appeared in two of the three runs; conversely, bands were scored as absent if two out of the three reactions yielded no band. Of the bands included in the study, >90% yielded the same result in all three PCR runs. These inclusion criteria helped to identify bands that were sensitive to reaction conditions.

**Table 4:** Genetic affinities in populations of the coral *Madracis decactis.*

(a)	*Madracis decactis*			
	Transects III & IV			
AFLPOP Analysis, Log-Likelihood = 0				
	**Percentage (%) of Colonies**			
	**W. of Miss. River**			**E. of Miss. River**
**Allocated to**	**ST-295**	**ST-297**	**ST-292**	**MP-All**
**ST-295**	100%	0	0.3	0
**ST-277**	0	100	0	0
**ST-292**	0.2	0	99.7	0
**MP - All**	0	0	0	100
**(b)**	**Log-Likelihood = 1**			
	**Percentage (%) of Colonies**			
	**W. of Miss. River**			**E. of Miss. River**
**Allocated to**	**ST-295**	**ST-297**	**ST-292**	**MP-All**
**ST-295**	99%	0	0	0
**ST-277**	0	100	0	0
**ST-292**	0.1	0	99.4	0
**MP - All**	0	0	0	100
**CNM**	0.6	0	0.6	0

(a) Genetic affinities on oil/gas platforms in the northern Gulf of Mexico along two transects – one south of Terrebonne Bay, LA (III), west of the Mississippi River mouth, and another south of Mobile, AL (IV), east of it. Data were combined in the Main Pass (MP) lease area due to small sample sizes per platform, to facilitate comparison. Data analyzed via AFLPOP, with a log-likelihood value set at 0. Note extraordinarily levels of high self-assignment to home populations and lack of recognition of neighboring populations. An indication of geographic isolation of these coral populations and a possible indication of different larval sources. (b) Same, but data analyzed with a log-likelihood value set at 1. Resulting pattern almost identical to analysis performed with more liberal 0 setting.

### Statistical Analyses

Two statistical analyses were used to assess population differentiation: AFLPOP population allocation analysis (V. 1.1; 73), a statistical analytical procedure designed particularly to analyze data generated by AFLPs, and STRUCTURE V 2.0 [74]. The development of these techniques and their application to the analysis of coral population genetics have been described elsewhere [57]–[59], [65], [66], [69]–[71], [75].

The AFLPOP analysis uses AFLP presence/absence data to calculate log-likelihood values for any individual’s membership in a reference population, based upon their banding patterns. The reference population is that target population (e.g., from one platform) against which all other colonies from other sites are compared for genetic affinity. Each individual is allocated to the population showing the highest likelihood for that genotype [64], [73]. These assignment tests have been successfully used to estimate long-distance dispersal [64]. When the individual is assigned to a population different than the site from which it was collected, it is interpreted as evidence of dispersal. One major advantage of the method is that populations do not have to be sampled exhaustively [64]. In an AFLPOP simulation, an individual is chosen randomly from the entire population, population marker frequencies are calculated without that individual, and then the individual is assigned to the new data set. This simulation was repeated 500 times for each run. Average assignments to a given site were subsequently calculated as a percent value, based on 10 repeats of these 500 iterations.

The AFLPOP program allows the user to set a log-likelihood threshold for each assignment. If set to 0.0, a colony will always be assigned to the population with the highest likelihood value. Atchison [57] and Atchison et al. [58], [59] found that this may yield misleading results. This is because individual assignment does not take into account that multiple sites may have nearly equal likelihood values. Here, we also performed simulations using 1.0 as the comparative log-likelihood threshold in the analysis. This approach is more conservative, though at the cost of some information. With the threshold set to 1.0, a colony would not be assigned to a population unless the probability of the given assignment was 10 times more likely than the next most probable assignment. If this threshold was not met, the individual was not assigned to any population and is designated “Criteria for allocation Not Met” (CNM). This does not imply that the sample could not be assigned to a population; it means that there may be at least two populations with nearly equivalent probabilities of assignment. It could also mean that the individual fits none of the populations well and may be derived from an outside population (see [58], [59]). In this study, we focused on cases where clear assignments have been made.

STRUCTURE uses Bayesian techniques and Monte Carlo simulations to assign samples to populations. Unlike AFLPOP, in which assignment is based solely upon marker frequencies, STRUCTURE makes assignments that minimize deviations from the Hardy-Weinberg (H-W) equilibrium, which assumes that the population giving rise to the recruits constitutes a large, randomly mating population. Using this approach, the program calculates probabilities of individual assignment, estimates of F_ST_, and probable paternity, grand-paternity, etc. relationships. The program can accommodate dominant marker data such as those generated by the AFLP technique. Data were analyzed using a burn-in period of 500,000 iterations, followed by another 100,000 Markov Chain Monte Carlo (MCMC) repetitions.

STRUCTURE also calls for definition of the parameter MIGPRIOR before running. This parameter was the prior probability of a spat being identified as coming from an external source. It was run at two levels, for comparative purposes – 0.05 and 0.50, taking into account different potential estimated migration rates.

We chose not to use AMOVA for our analysis. Although AFLP data can be treated like allelic data, since these markers are co-dominant and multi-locus, we chose to take a more conservative approach by using genetic assignment tests. Genetic assignment tests are also more appropriate to the types of questions we have addressed here. Finally, AFLP data can be problematic in attempting to estimate “allele” frequency distributions among populations, plus classic parameters like *Nm* and *F_ST_* can in themselves be problematic as indirect genetic indices of assessing population connectivity see [76]–[79]).

**Figure 4:**
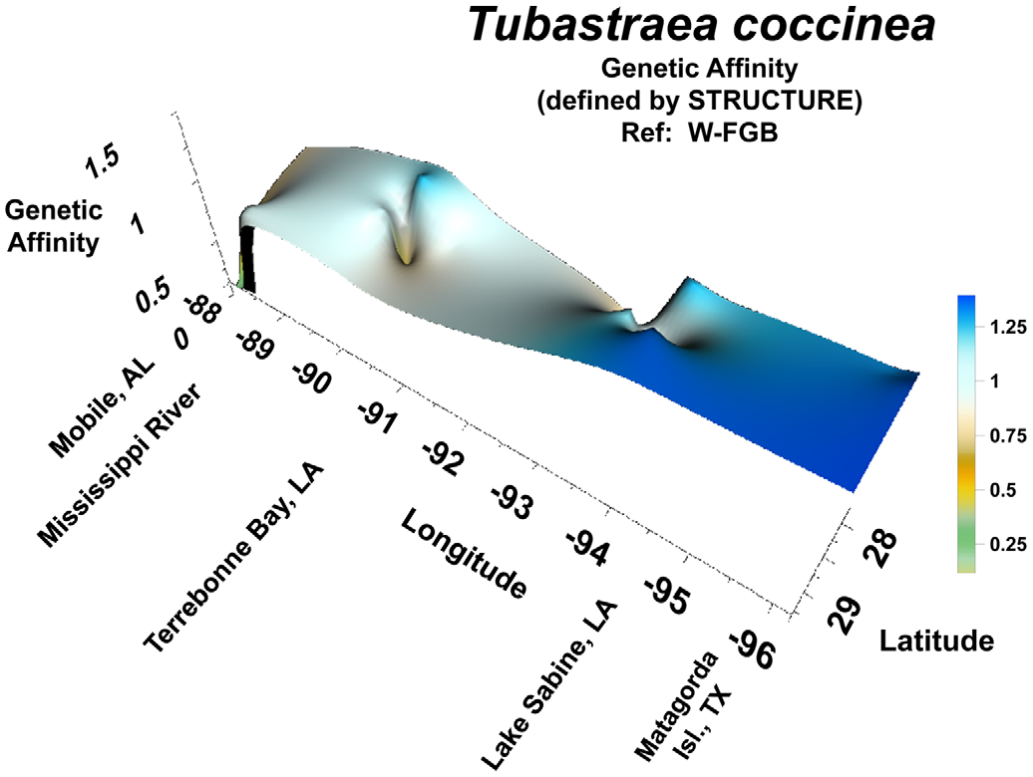
Genetic affinity in *Tubastraea coccinea* coral populations on oil and gas platforms across the continental shelf in the northern Gulf of Mexico. Genetic affinity value (degree of relatedness) determined by the population genetic analytical software STRUCTURE. The reference population was the West Flower Garden Bank; *i.e.,* the population against which all other members of all other populations were compared. The relative flatness of the curve indicates no major local larval source; i.e., it is unlikely that the FGB are a source of larvae for the region for this species. The steep decline in genetic affinity in the east indicates major population differences between the two sides of the Mississippi River. The point depression south of Terrebonne Bay indicates a population from a very different source than the rest of the western populations. Note: The orientation of the map has been reversed to east-to-west in order to facilitate viewing of the topography of the three-dimensional pattern generated by the data. This reveals fine-scale structure that would otherwise be hidden using a southerly view. The reader is viewing the region from the north, with east being on the left and west on the right.

**Table 5:** Genetic affinities in populations of the coral *Tubastraea coccinea.*

a.		*Tubastraea coccinea*				
		Transects III & IV				
	**AFLPOP Analysis - Log-Likelihood = 0**					
	**Percentage (%) of Colonies on these Platforms**					
	**W. of Miss. River**	**E. of Miss. River**				
**Allocated to**	**ST & SS pops**	**MP-144**	**MP-236**	**MP-265**	**MP-288**	**MP-289**
**ST & SS pops**	**100%**	0	0	0	0	0
**MP-144**	0	**70**	0.1	8.6	4.2	9.3
**MP-236**	0	0.6	**99.7**	0.2	0	0.5
**MP-265**	0	12.6	0.1	**90.3**	0.4	0.6
**MP-288**	0	4.6	0	0.3	**94.8**	0.8
**MP-289**	0	12.2	0.1	0.6	0.6	**88.8**
			N_i_ - 500, 10x			
**b.**	**Log-Likelihood = 1**					
	**Percentage (%) of Colonies on these Platforms**					
	**W. of Miss. River**	**E. of Miss. River**				
**Allocated to**	**ST & SS pops**	**MP-144**	**MP-236**	**MP-265**	**MP-288**	**MP-289**
**ST & SS pops**	**100%**	0	0	0	0	0
**MP-144**	0	**21.4**	0	0.8	0.2	0.4
**MP-236**	0	0.4	**98.4**	0	0	0
**MP-265**	0	1.6	0	**58.6**	0	0
**MP-288**	0	1	0	0	**82.8**	0.2
**MP-289**	0	2.2	0	0	0	**57.8**
**CNM**	0	73.4	1.6	40.6	17	41.6
			N_i_ - 500, 10x			

(a) Genetic affinities on oil/gas platforms in the northern Gulf of Mexico along two transects – one south of Terrebonne Bay, LA (III), west of the Mississippi River mouth, and another south of Mobile, AL (IV), east of it. Platforms on the western side combined to provide sufficient sample size for comparison. Data analyzed via AFLPOP, with a log-likelihood value set at 0. (b) Same, but using a log-likelihood value of 1.0. Note how self-assignment arguments have diminished in magnitude, indicating much greater levels of cross-assignment, varying greatly from analysis performed with more liberal 0 setting. This indicates a broader dispersal capacity for *T. coccinea vs. M. decactis* (see Table 4).

**Figure 5:**
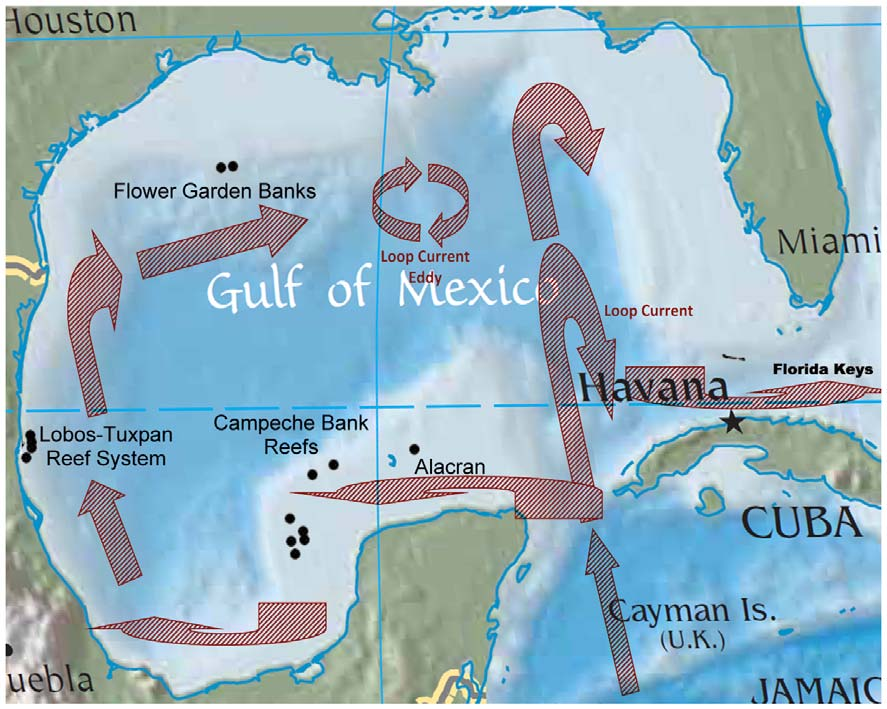
Map of the Gulf of Mexico, depicting examples of general known currents. Note the general westerly current along the edge of the continental shelf in the vicinity of the Flower Garden Banks.

## Results

### Relationship between Genetic Distance and Geographical Distance for *Madracis decactis*

In examining *Madracis decactis*, the results derived from STRUCTURE revealed a clear pattern. The highest levels of genetic affinity occurred between coral populations on platforms in Transect II, off of Port Arthur/Lake Sabine, TX, and on platforms around the FGB (Fig. 3). This affinity peaked in the west and then decreased steadily and to the east, to Transect III, south of Terrebonne Bay. Affinity then increased slightly in Transect IV, off Mobile, AL. A point depression in genetic affinity occurred near the edge of the continental shelf in Transect III (off Terrebonne Bay, LA).

We compared genetic affinities of *Madracis decactis* populations between the two sides of the Mississippi River mouth to determine whether the river plume – a major oceanographic feature - could be acting as a geographic barrier to dispersal. When analyzing the genetic data using AFLPOP, with a log-likelihood threshold value of “0” (all colonies must be assigned to a population), it became clear that not only was there little similarity between the two populations, there was also little similarity among platform populations within a transect (Table 4a). When this analysis was repeated using a log-likelihood threshold value of 1.0 (a colony must be 10X more likely to belong to a given population than another before being assigned there), a much more conservative approach, there was still very little difference in the results (Table 4b). Both analyses indicated minimal levels of dispersal between these two sets of populations – between or within transects.

### Relationship between Genetic Distance and Geographical Distance for *Tubastraea coccinea*

The analytical program STRUCTURE yielded clear results for *Tubastraea coccinea*. In general, the genetic affinity values in this species were higher across the continental shelf than those for *Madracis decactis* (Fig. 4; see Y-axis). Although genetic affinity generally decreased from west to east, there was substantial local variation in this pattern. Firstly, genetic affinities dropped highly significantly east of the Mississippi River, in Transect IV. In addition, there was an anomalous point depression in genetic affinity in *T. coccinea* at the edge of the continental shelf in Transect III (off Terrebonne Bay, LA).

When one makes a direct comparison of genetic affinities of populations occurring in Transect III (off Terrebonne Bay, LA) *vs*. Transect IV (off Mobile, AL) using AFLPOP with a log-likelihood threshold value of 0.0 (forcing assignment), there was once again little similarity anong populations across the mouth of the Mississippi River (Table 5a). A higher degree of recognition occurred between populations of Madracis decactis within these transects. When this analysis was repeated using a log-likelihood threshold value of 1.0 (more conservative), self-assignment to home populations decreased and more colonies were assigned to the CNM (Criteria Not Met) category (Table 5b). In general, dispersal appeared to be broader in *Tubastraea coccinea* than in *Madracis decactis*.

## Discussion

The high degree of connectivity between populations of *Madracis decactis* on platforms in the western GOM, as determined by STRUCTURE, confirms that these populations most likely originated from the Flower Garden Banks (FGB). The platform populations show high affinity for those on the FGB and those on platforms around them. Also, as the platform populations become more distant from those in the west and the FGB, they exhibit less genetic affinity to each other. The slight increase in genetic affinity in the eastern sector, beyond the mouth of the Mississippi River mouth, underscores the lack of affinity between the populations on the eastern and western sides of the river. The mouth of the Mississippi River appears to represent a strong geographic barrier to larval dispersal, as is known to be the case in other organisms in the vicinity of river mouths, particularly the Mississippi (e.g. bivalves and other organisms; see [80]–[84]) because of its physical and chemical characteristics. Success of fertilization of eggs is known to be affected by low salinities [85], [86], as is planular development, settlement, and survival [87]; (but see [88]. Other related factors affection planular survival and settlement are sedimentation [89) and increased nutrients [90], [91].

The anomalous point depression in genetic affinity observed in *Madracis decactis* in Transect II, off Terrebonne Bay, LA suggests that some *Madracis decactis* larvae may have been introduced to this region by more than one means – from more than one region - or more than one time. In the first case, larvae may have been introduced from the Caribbean via the Loop Current entering the Gulf of Mexico through the Yucatan Straits, from the Caribbean, proceeding north to the Mobile, AL region (Transect IV; Fig. 5). Alternatively, *Madracis decactis* larvae may have been introduced via a jet current from the northern portion of the Yucatan Peninsula [92]. In addition, hurricanes can promote larval dispersal over long distances by producing high speed currents that could move larvae from the Caribbean to the northern GOM [93]. In any case, it is clear that this *Madracis* population is different from all other *M. decactis* populations sampled in the northern GOM.

The geographic pattern of genetic affinity exhibited by *Tubastraea coccinea* (as determined by STRUCTURE) had general similarities to that of *Madracis decactis*; that is, there was a general decrease in affinity from west to east. The variations in this pattern observed in *M. decactis*, however, were much more striking in *T. coccinea*. Firstly, the strong decrease in genetic affinity near the mouth of the Mississippi River indicates a sharp differentiation between that far-eastern population and all of those to the west of the Mississippi River mouth. In addition, a deep point depression in genetic affinity of *T. coccinea* was also noted in Transect II. It was similar to that observed in *Madracis decactis*, but more marked. The population on that platform, was clearly unrelated to the others in the northern GOM. This may represent a second introduction of this species to the northern Gulf of Mexico. Potential sources include the Caribbean, as described above, or a second successful introduction to the western Atlantic from a commercial vessel from the Indo-Pacific. Once again, in either case, this population is unrelated to the others in this region.

The Mississippi River appears to represent a formidable east-west barrier to coral larval dispersal in this region. This was evidenced through the lack of genetic affinity between coral populations on either side. This pattern was evident in two distantly related coral species - *Madracis decactis* and *Tubastraea coccinea*.

There are two possible explanations for this anomaly which are not mutually exclusive. The first is that the lower salinities, higher sediment, and higher nutrient concentrations associated with the Mississippi River plume [94]–[97] may decrease coral larval survivorship levels in this region [90], [91]. This would result in increased geographic isolation of the coral populations. The second is that coral larvae from the Caribbean may be derived from elsewhere, carrying with them a different genetic identity. Larvae could have been transported north by the Caribbean Current into the northern Gulf of Mexico via the Loop Current, major oceanographic feature in this region. Eddies that break off from the Loop Current can traverse the Main Pass (MP) region (near eastern sector, Transect IV), prior to moving W-SW over the next 6 mos or so (see [98] for an overview of these features).

Our results also revealed that the invasive ahermatypic *Tubastraea coccinea* has higher larval dispersal and recruitment capabilities than *Madracis decactis*. The latter species is clearly the most abundant and widely dispersed native hermatypic species on the platforms,yet its populations showed almost no genetic affinity to each other when compared across the two sides of the Mississippi River mouth. In addition, the populations showed little if any affinity to each other within a transect on a given side. Populations of *T. coccinea*, although exhibiting a similar high degree of separation across the river mouth, showed more recognition between platforms within a transect – suggesting higher dispersal between them.

These results support the findings from our earlier study [25], [58], [69] - that populations of *Madracis decactis* and other corals are highly isolated on these platforms and that their variation from each other most likely results from Founder Effect (see [99] for a discussion of similar patterns in deep-sea corals). This phenomenon is a by-product of colonization by a small number of larvae – a sample-size effect. That is, the local population resulted from arrival of a small sub-group of larvae from the mother FGB population, carrying with it a genetic identity specific to this sub-group. With time, the population continued to grow in size, possibly experiencing genetic drift, to produce a larger sub-population with a different genetic signature [100], [101].

The higher dispersal rates exhibited by *Tubastraea coccinea* may be one of the major reasons this species has been so successful in its distribution throughout the Greater Caribbean region since its introduction 70 yrs ago. Its reproductive, larval dispersal, and recruitment capabilities are high – higher than our dominant native species. Its dispersal capabilities are also better than those of one of the only other successfully introduced coral species – *Fungia scutaria* [41]–[43]. Indeed, with reproductive characteristics like these (including rapid asexual reproduction and growth), the only reason that this species has not over-run our natural coral reefs is that it apparently does not compete well for space in natural Caribbean systems – only in artificial habitats (breakwaters, platforms, bridge pilings, etc.).

The second inference that may be drawn from these genetic affinity patterns is that some other Indo-Pacific species may be similarly better adapted to reproduce and disperse than our native species, and that all precautions should be taken to eliminate them should they be successfully introduced in our waters. Sammarco et al. [44] recently reported the new introduction of a closely related species – *Tubastraea micranthus* – into the northern GOM in the Grand Isle offshore oil/gas lease area – S-SW of the mouth of the Mississippi River. This area borders on commercial shipping lanes (safety fairways) leading to Port Fourchon, and New Orleans *via* the Mississippi River. We are currently attempting to determine the extent of the invasion [102]. Nonetheless, a rapid eradication should be considered for such species because of the possibility of success of such an action if taken early [103], the decreasing probability of success of eradication with time [104], [105], and major problems that can arise if one waits far too long to proceed with eradication [106], [107].
